# The Congress on School Hygiene

**Published:** 1913-08

**Authors:** 


### BUFFALO MEDICAL JOURNAL

**A Monthly Review of Medicine and Surgery**

EDITOR AND PUBLISHER DR. A. L. BENEDICT, 228 Summer St., corner of Elmwood Ave., Buffalo. (Address for all communications. Please make personal and telephone calls before 1 P. M.)

THE BUFFALO MEDICAL JOURNAL is the third, in age, in the western continent. It is independent but supports professional organization. As a scientific medium it is unrestricted in territory. As a news medium, it is limited mainly to the western four districts of the Medical Society of the State of New York. The co-operation of physicians is asked in securing personal, obituary and other news items. Secretaries of Medical Societies in this region are requested to send brief reports of meetings. Full transactions will be published at cost of composition.

Subscription $2.00 a year in advance; $3.00 for two years in advance. Changes and errors in address or failure or duplication of delivery, should be reported immediately. Back numbers will be supplied, if possible but requests for copies should be made promptly,

YEARLY VOLUME 69 AUGUST, 1913 No. 1

*The child is father to the man. Let us be sure that the future adult has a healthy, moral and intelligent parent.*

### The Congress on School Hygiene

We welcome to our city this body of distinguished philanthropists and educators and bespeak the interest in the cause of the medical profession generally, not only in attendance at the meetings but in permanent support of the various lines of activity which the Congress will initiate or stimulate. It would be so easy to anticipate the general trend of the papers and to recapitulate methods and aspirations already familiar to our readers, that we forbear to do so. Rather let us offer two suggestions of conservatism, by no means in a spirit of opposition: Do not let the child become too much impressed with his own importance, especially to the extent of feeling that he is the main object of adult labor and provision and that he is himself free from the responsibility of industry. And, in utilizing to the full, the School as an institution where practically the entire child population can be reached and benefited, do not forget the paramount importance of the home, nor let the school usurp the responsibilities of parenthood.

### Protect the Little Boy

We commend to individuals and societies especially interested in conserving the morals of the nation, a factor often overlooked —the innocence and purity of the little boy. We are not attempting to upset the world-wide and world-old notion that

